# A phylogenomic framework for assessing the global emergence and evolution of clonal complex 398 methicillin-resistant *Staphylococcus aureus*

**DOI:** 10.1099/mgen.0.000105

**Published:** 2017-01-31

**Authors:** Anders Gonçalves da Silva, Sarah L. Baines, Glen P. Carter, Helen Heffernan, Nigel P. French, Xiaoyun Ren, Torsten Seemann, Dieter Bulach, Jason Kwong, Timothy P. Stinear, Benjamin P. Howden, Deborah A. Williamson

**Affiliations:** ^1^​ Doherty Applied Microbial Genomics, Department of Microbiology & Immunology, The University of Melbourne at The Doherty Institute for Infection and Immunity, Melbourne, Australia; ^2^​ Microbiological Diagnostic Unit Public Health Laboratory, Department of Microbiology & Immunology, The University of Melbourne at The Doherty Institute for Infection and Immunity, Melbourne, Australia; ^3^​ Institute of Environmental Science and Research, Wellington, New Zealand; ^4^​ Infectious Disease Research Centre, Massey University, Palmerston North, New Zealand; ^5^​ Victorian Life Sciences Computation Initiative, Melbourne, Australia

**Keywords:** Livestock-MRSA, swine, epidemiology, *Staphylococcus aureus*, zoonosis

## Abstract

Distinct clones of methicillin-resistant *Staphylococcus aureus* (MRSA) have emerged as important causes of infection in individuals who have exposure to livestock (livestock-associated MRSA; LA-MRSA). Clonal complex 398 (CC398) is the most prevalent LA-MRSA clone, and has been reported from several geographical settings, including Europe, the Americas and Asia. To understand the factors contributing to the global dissemination of this clone, we analysed CC398 MRSA isolates from New Zealand (NZ), a geographically isolated country with an economy strongly dependent on livestock farming. We supplemented the NZ CC398 MRSA collection with global datasets of CC398 MRSA and CC398 methicillin-susceptible *S. aureus.* Here, we demonstrate multiple sporadic incursions of CC398 MRSA into NZ, as well as recent importation and spread of a swine-associated clade related to the European LA-MRSA lineage. Within a larger global phylogenomic framework, Bayesian modelling suggested that this NZ clade emerged in the late 2000s, with a probable origin in swine from Western Europe. Elucidating the factors responsible for the incursion and spread of LA-MRSA in geographically distant regions, such as NZ, provides important insights into global pathways of *S. aureus* transmission, and will inform strategies to control importation and spread.

## Abbreviations

CC398, clonal complex 398; HPD, highest posterior density; LA-MRSA, livestock-associated methicillin-resistant Staphylococcus aureus; MGE, mobile genetic element; ML, maximum-likelihood; MRCA, most recent common ancestor; MRSA, methicillin-resistant Staphylococcus aureus; NZ, New Zealand; SCC, staphylococcal cassette chromosome.

## Data Summary

Genome NZ15MR0322 has been deposited in the European Nucleotide Archive (ENA); Project number: PRJEB12552 www.ebi.ac.uk/ena/data/view/PRJEB12552.

## Impact Statement

The emergence and spread of a specific livestock-associated lineage of methicillin-resistant *Staphylococcus aureus* [clonal complex 398 (CC398) MRSA] has been well described in Europe and North America. However, little is known about the circulation of CC398 MRSA in the southern hemisphere. Here, we report the emergence and evolution of CC398 MRSA in humans in New Zealand (NZ), a geographically remote country with more livestock than people. A distinct NZ sub-lineage of CC398 MRSA was identified that was epidemiologically associated with exposure to swine in NZ. Analysis suggested this NZ clade emerged in the late 2000s, with a probable origin in swine from Western Europe. Our findings provide an explanation for the introduction and spread of CC398 MRSA in NZ with implications for disease control.

## Introduction

*Staphylococcus aureus* is a major pathogen in both human and animal populations. Distinct clones of methicillin-resistant *S. aureus* (MRSA) have emerged as important causes of infection in individuals who have direct exposure to livestock (livestock-associated MRSA; LA-MRSA) [1]. Clonal complex 398 (CC398) MRSA is the most prevalent LA-MRSA clone, and has been reported from a range of geographical locations, including Europe, the Americas and Asia [2, 3]. Discrete genetic clades of CC398 have been described, varying predominantly in phage-related mobile genetic elements (MGEs), and their association with different hosts and geographical location [3–5]. In particular, genes belonging to the staphylococcal immune evasion cluster (staphylococcal complement inhibitor, *scn*; staphylokinase, *sak*; staphylococcal enterotoxin A, *sea*; and chemotaxis inhibitory protein, *chp*) are generally associated with CC398 *S. aureus* recovered from humans [6, 7], and genes encoding tetracycline resistance [*tet*(M)], methicillin resistance (*mecA*) and trimethoprim resistance (*dfrG*) have been predominantly associated with CC398 *S. aureus* recovered from livestock [3]. Moreover, it has been observed that isolates from livestock and human clades may be broadly differentiated based on three canonical SNPs [8].

Despite improved understanding of the prevalence and transmission of CC398 *S. aureus* in Europe and North America, little is known about the circulation of CC398 *S. aureus* in the southern hemisphere. This is particularly important in New Zealand (NZ), a country with a sizeable agricultural industry and a large livestock population. CC398 MRSA was first isolated in NZ in 2011, when four patients with CC398 MRSA infection were identified [9]. Given the strong association between livestock exposure and CC398 MRSA isolation in other settings, knowledge of the clinical and genomic factors that may facilitate the emergence and spread of CC398 MRSA in NZ is critical. In this context, the specific aim of this study was to characterize the phylogeographical context, evolution and putative origins of CC398 MRSA in NZ, in order to understand the factors that may contribute to, and mitigate, the global dissemination of this clone.

## Methods

### Setting and isolate collection

NZ is an island nation located in the South West Pacific, with a population of approximately 4.47 million. In August of each year, a national survey of MRSA is performed, where all NZ community and hospital laboratories refer consecutive, non-duplicate MRSA isolates to the Institute of Environmental Science and Research (ESR) for molecular typing. In addition, throughout the year, laboratories also refer MRSA isolates to ESR with unusual antimicrobial resistance profiles, or isolates associated with outbreaks for further characterization. As part of these active and passive surveillance systems, 36 CC398 MRSA were identified from 36 patients (24 from clinical specimens; 12 from screening) between January 2011 and September 2015 (Supplementary Dataset).

### Genome sequencing and genomic analysis

Genomic DNA extraction, library preparation and Illumina sequencing were performed as previously described [10]. CC398 MRSA isolate NZ15MR0322 was sequenced on the RS-II platform (Pacific Biosciences) using SMRT technology. Genomic and phylogenetic analyses were performed as described in Supplementary Text S1. All sequence data are available at the European Nucleotide Archive (ENA) under BioProject PRJE12552.

### Ethics

Given the low-risk observational nature of this study, formal ethical review was not deemed necessary by the New Zealand Health and Disability Ethic Committees (Ref: 15/STH/164).

## Results

### Complete genome sequence of NZ15MR0322 ST398 MRSA

To further characterize CC398 MRSA from NZ, and provide a high-quality reference genome for subsequent genomic analysis, a representative strain was sequenced and completely assembled. This strain was isolated in 2015 from a patient with a superficial skin infection. The genome of NZ15MR0322 comprised a circular chromosome of 2 839 206 bp (G+C content of 32.9 mol%) and a single circular plasmid (pNZ15MR0322) of 27 186 bp (G+C content of 29.9 mol%). *In silico* multilocus sequence typing confirmed that this isolate belonged to ST398. Similar to previously described plasmids in ST398 MRSA [11], plasmid pNZ15MR0322 contained genes associated with both cadmium resistance (*cadD* and *cadX*) and copper resistance (*copA* and *mco*), but harboured no other known resistance or virulence determinants.

**Fig. 1.**
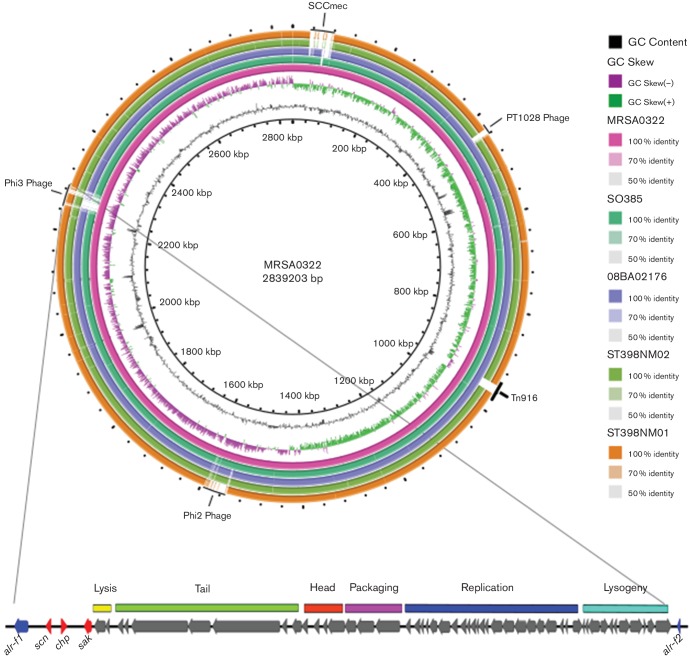
Sequence comparison of representative genomes of CC398 *S*. *aureus*, illustrating a high level of sequence conservation amongst the CC398 lineage. Genomic regions in NZ15MR0322 that were variably present in other CC398 *S. aureus* include the SCC*mec*V mobile element, and two complete phage regions (ϕSa2 and ϕSa3).

A number of MGEs were present within the chromosome of NZ15MR0322, including a type Vc (5C&5) staphylococcal cassette chromosome (SCC) *mec* element previously described in CC398 MRSA from the European LA-MRSA lineage [2] (Fig. 1). Two complete prophages were detected, belonging to the ϕSa2 and ϕSa3 families, respectively. Based on high identity blast matches, the 46.9 kb ϕSa2 most closely resembled ϕStauST398-2 (GenBank accession no. JQ957932), with 92.7 % nucleotide sequence identity (Fig. 1). The 42.6 kb ϕSa3 in NZ15MR0322 contained functional modules sharing regions of similarity with other ϕSa3, including genes associated with immune evasion in humans (*scn*, *chp* and *sak*). Unlike typical ϕSa3, which are generally integrated into the beta-haemolysin (*hlb*) gene, the ϕSa3 in NZ15MR0322 was instead integrated into the alanine racemase (*alr*) gene (Fig. 1), approximately 193.5 kb downstream of an intact *hlb* gene. Alanine racemase is an enzyme that catalyses reversible racemization between l-alanine and d-alanine (an essential cell-wall component). The insertion of ϕSa3 split *alr* into two discrete fragments (Fig. 1). Previous work has suggested that the acquisition of this prophage in CC398 LA-MRSA may be associated with ‘re-adaptation’ to humans [7], and may facilitate sustained human-to-human transmission of CC398 LA-MRSA, an observation currently rare within this clone. It is therefore of concern that a recent NZ CC398 MRSA, clustering with isolates associated with livestock or livestock exposure, has apparently re-acquired an MGE associated with human immune evasion, and suggests possible evolutionary adaptation to human infection within NZ.

### Phylogeographical context and comparative genomics of NZ CC398 MRSA

**Fig. 2.**
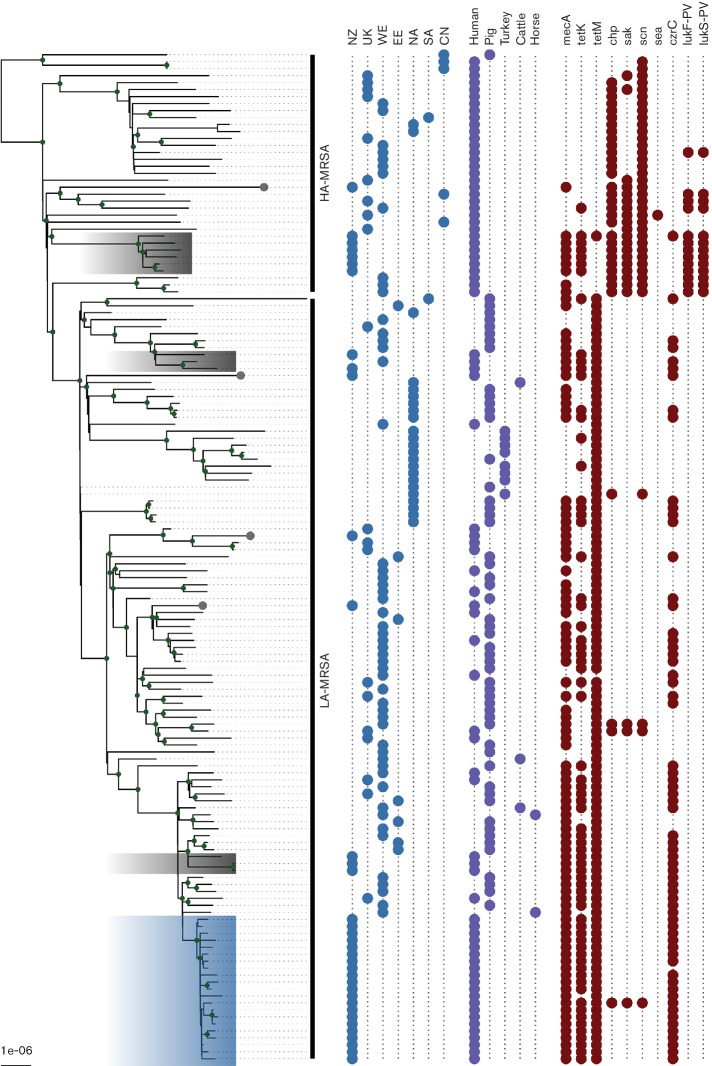
Population structure of CC398 *S. aureus*. The ML tree, reconstructed from 6654 core genome SNPs, illustrates the population structure of the representative global population of CC398 *S. aureus.* NZ, New Zealand; SA, South America; EE, Eastern Europe; WE, Western Europe; CN, China; UK, United Kingdom; HA-MRSA, human-associated MRSA; LA-MRSA, livestock-associated MRSA. The blue shading indicates the NZ CC398 MRSA sublineage, and grey shading/tips indicate NZ isolates.

To characterize the population structure of CC398 MRSA in NZ, all 36 NZ isolates underwent whole genome sequencing. The NZ collection was supplemented with reads from 56 CC398 MRSA and 50 CC398 methicillin-susceptible *S. aureus* globally representative isolates from previously published studies (Supplementary Dataset) [3, 5]. Mapping of reads to reference strain NZ15MR0322 identified 25 358 single nucleotide variants, and after removal of recombinogenic sites, there were 6654 SNPs present within the core genome. These core genome SNPs were used to reconstruct the phylogenetic relationship among the sampled isolates using a maximum-ikelihood (ML) approach (Fig. 2).

In keeping with previous findings [3, 5], the ML tree clustered isolates into two main clades: one associated predominantly with CC398 methicillin-susceptible *S. aureus* from humans and the other associated predominantly with CC398 MRSA from livestock and environmental samples (Fig. 2). Of the 36 NZ isolates, 21 clustered within a distinct NZ CC398 MRSA lineage in the larger LA-MRSA clade, with a median core genome pairwise SNP distance between isolates in this clade of only 16 SNPs (Fig. 2). Of the 21 isolates, 18 were from patients in the South Island of NZ. Although occupational information was not available for all patients, based on information provided to the laboratory, 10 of the 18 patients had either direct exposure, or indirect household exposure, to swine farming or meat production in NZ.

The clinical epidemiological association of swine and patients in NZ with CC398 MRSA isolates was supported by results in the ML tree. First, the NZ sublineage formed part of a larger clade previously associated with European CC398 LA-MRSA isolates, predominantly from swine [3]. Second, similar to other CC398 LA-MRSA from swine [3], most isolates within this larger clade (including those sampled in NZ) contained the *tet*(M) gene, and harboured a type V (5C&5) SCC*mec* element (Fig. 2). Finally, none of the strains within the NZ sublineage contained canonical SNPs previously associated with CC398 *S. aureus* of human origin [8]. In addition to the discrete NZ CC398 MRSA lineage, CC398 MRSA isolates from 15 other patients in NZ were distributed throughout the ML tree, including isolates that clustered with human-associated CC398, consistent with multiple independent introductions of CC398 MRSA into NZ (Fig. 2) [3].

### Spatiotemporal and host evolutionary origins of CC398 MRSA in NZ

**Fig. 3.**
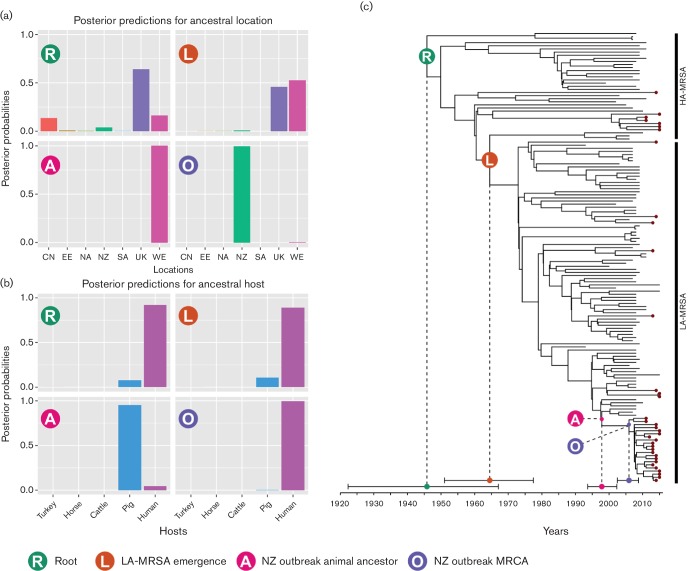
Bayesian evolutionary analysis demonstrating the timing of divergence within the CC398 clade, as well as the most likely location and host for a subset of four relevant nodes: R, the MRCA of all CC398 samples; L, the MRCA of LA-MRSA; A, the non-human ancestor of the most recent NZ LA-MRSA outbreak; O, the human MRCA of the most recent NZ LA-MRSA outbreak. (a) Posterior probability mass distribution of geographical locations for four highlighted nodes. (b) Posterior probability mass distribution of host species for four highlighted nodes. (c) Maximum clade credibility genealogy for CC398 samples, depicting the median and 95 % HPD for age of the four important nodes. Isolates from NZ are labelled with a burgundy tip. NZ, New Zealand; SA, South America; EE, Eastern Europe; WE, Western Europe; CN, China; UK, United Kingdom; HA-MRSA, human-associated MRSA.

To further investigate the evolutionary history of the NZ CC398 MRSA lineage, and identify potential ancestral host and geographical reservoirs, a phylogenetic reconstruction of the NZ CC398 lineage was performed using beast2 [12], within the framework of the larger dataset [3, 5]. Bayesian analysis estimated the rate of nucleotide substitutions for the CC398 clone at a median 1.42×10^−6^ substitutions per site per year (ss^−1^ yr^−1^) [95 % highest posterior density (HPD) interval 1.023×10^–6^–1.808×10^−6^ ss^−1^ yr^−1^], similar to 95 % HPD interval estimates from other *S. aureus* clones [13, 14] This estimate places the date of the most recent common ancestor (MRCA) of the NZ CC398 MRSA lineage to be 2005 (95 % HPD interval 2002–2008), consistent with the first known isolations of this lineage in NZ in 2011 (Fig. 3) [9].

To explore the possible geographical origins of NZ CC398 isolates, we performed a phylogeographical analysis using beast2, employing an adaptation of the model described by Ward *et al.* [5]. To reduce the number of parameters in the model, we grouped samples into seven geographical regions: NZ, UK, Western Europe, Eastern Europe, North America, South America and China. To explore putative host switching in CC398, we also added the sampled host as a discrete trait to the model. Five possible hosts were included: human, pig, cattle, horse and turkey. In addition, to further reduce the model parameters, we used an HKY substitution model, and included only core SNPs in a single partition. A comparison with parameter estimates and topology to those obtained with the full model above demonstrated the equivalence of the results. The 95 % posterior credible set for the root geographical and host origins of the NZ CC398 MRSA lineage strongly supported a putative origin for the NZ clade from Western Europe, with swine emerging as the likely ancestral host (Fig. 3 – tree node A). However, an important limitation of this work, and indeed of other phylogenomic studies of CC398 [3, 5], is non-uniform sampling of hosts and geographical regions. Additional broader and deeper sampling is required to investigate other potential origins and hubs of dissemination. For example, denser sampling in under-represented regions such as Africa or Asia may identify other regionally important hubs.

## Discussion

Collectively, our analyses support the existence of two concurrent pathways for the emergence of CC398 in NZ. The first is due to multiple sporadic introductions of CC398 MRSA into NZ from a range of geographical locations, with no extensive onward transmission between humans. The second, more concerning, pathway is probably due to the recent importation of an isolate related to the European LA-MRSA lineage, and the relatively high number of isolates from patients with swine exposure suggests the possibility of a swine reservoir in NZ, with resultant ‘spillover’ into humans. Supporting the European origin of the NZ sublineage is clustering within the larger European LA-MRSA clade, and regions of high homology to MGEs (e.g. SCC*mec*) within the European lineage. Moreover, our Bayesian framework suggests importation of this sublineage within the past decade, with a possible origin in swine from Western Europe.

Although NZ has relatively low rates of agricultural antimicrobial use compared to other developed countries [15], recent data on the use of antimicrobials in food animals in NZ demonstrated that tetracycline sales approximately doubled from ∼4.00 tonnes per year in 2005 to 7.99 tonnes per year in 2014 [15]. In NZ, as in other settings, the major indications for agricultural tetracycline use are in the pig and poultry industries [16]. It is therefore possible that the increased use of tetracyclines in livestock is a potential contributor to the emergence and spread of CC398 MRSA in NZ. To date, however, there has been no systematic sampling of swine to assess the prevalence of CC398 MRSA in swine herds in NZ. To mitigate the spread of this clone, future work such as a case-control study should attempt to determine the risk factors for CC398 MRSA acquisition in NZ.

## Conclusion

We have described the recent emergence and evolution of CC398 MRSA in a geographically isolated region, within the context of a large and globally diverse dataset. To our knowledge, this is the first detailed phylogenomic analysis of CC398 MRSA from Oceania, and the combination of our genomic models allowed us to build a hypothesis for the origin and spread of CC398 MRSA in NZ. Elucidating the factors responsible for the invasion and spread of LA-MRSA in geographically distant regions, such as NZ, will provide important knowledge into global pathways of *S. aureus* transmission.

## Data Bibliography

1. Goncalves Da Silva A, Baines SL, Carter GP, Heffernan H, French NP *et al*. Sequence Read Archive BioProject PRJEB12552 (2017).
2. Uhlemann AC, Porcella SF, Trivedi S, Sullivan SB, Hafer C *et al*. Identification of a highly transmissible animal-independent *Staphylococcus aureus* ST398 clone with distinct genomic and cell adhesion properties. *MBio* 2012;3:e00027-12. GenBank Accession: PRJNA66999 and GenBank Accession: AIDT01000001.1.
3. Schijffelen MJ, Boel CH, Van Strijp JA, Fluit AC. Whole genome analysis of a livestock-associated methicillin-resistant *Staphylococcus aureus* ST398 isolate from a case of human endocarditis. *BMC Genomics* 2010;11:376. BioProject: PRJNA224116.
4. Price LB, Stegger M, Hasman H, Aziz M, Larsen J *et al.* *Staphylococcus aureus* CC398: host adaptation and emergence of methicillin resistance in livestock. *MBio* 2012;3:e00305-11. BioProject: PRJNA274898.
5. Ward MJ, Gibbons CL, Mcadam PR, Van Bunnik BA, Girvan EK *et al.* Time-Scaled Evolutionary Analysis of the Transmission and Antibiotic Resistance Dynamics of *Staphylococcus aureus* Clonal Complex 398. *Appl Environ Microbiol* 2014;80:7275–7282. BioProject: PRJEB7209.
6. Hadjirin NF, Lay EM, Paterson GK, Harrison EM, Peacock SJ *et al.* Detection of livestock-associated meticillin-resistant *Staphylococcus aureus* CC398 in retail pork, United Kingdom, February 2015. *Euro Surveill* 2015;20(24). European short read archive accession numbers ERR902083, ERR902084 and ERR902085.

## Supplementary Data

61 Supplementary File 1

## Supplementary Data

61 Supplementary File 2
