# Supplementary material for: A phylogenomic framework for assessing the global emergence and evolution of clonal complex 398 methicillin-resistant Staphylococcus aureus
Source: Microb Genom. 2017 Jan 31;3(1):e000105. doi: 10.1099/mgen.0.000105 (PMC5361625; doi:10.1099/mgen.0.000105)
Supplement: Supplementary File 1 [file mgen-3-105-s001.pdf]

Supplementary Text S1

Genome sequencing and comparative genomic analysis

CC398 MRSA isolate NZ15MR0322 was sequenced on the RS-II platform (Pacific Biosciences) using SMRT® technology, with library enriched for >10kb fragments. CC398 sequence reads were mapped to reference strain MRS150322, and single nucleotide polymorphisms (SNPs) were detected using *Snippy* (version 2.9; https://github.com/tseemann/snippy), with a minimum mapping coverage threshold of 10 and minimum 90% frequency of alternate allele. Illumina sequence reads were assembled using SPAdes (Bankevich et al., 2012) and PacBio sequence reads were assembled using the Hierarchical Genome Assembly Process HGAP (Agnoletti et al., 2014). Contigs were annotated using Prokka (Seemann, 2014), and multilocus sequence typing (MLST) was performed *in silico* using the MLST tool (version 2.4 – https://github.com/tseemann/mlst). Phage sequences were identified using PHAST (http://phast.wishartlab.com), and manually inspected for consistency. BRIG was used to compare the genome content of ST398 isolates (Alikhan et al., 2011). BLASTN searches were performed on *de novo* contigs using an in-house Python script, fastablasta (https://github.com/kwongj/fastablasta). The presence or absence of genes was determined using thresholds of 90% nucleotide identity and 90% coverage of the query sequence length. We searched for the presence of the following genes associated with antimicrobial resistance and host specificity: *mecA* (CP003166), *czrC* (AM990992), *lukF-PV* and *lukS-PV* (CP003166 and CP003166, respectively), *tetM* (AM990992), *tetK* (NC_017334), *Sa3Int* (BX571857), and the IEC genes *scn, sak, chp,* and *sea* (BX571857; BX571857: BX571857; and BX571857, respectively).

Phylogenomic analysis

Whole-genome short-read sequence data from 145 *S. aureus* isolates was trimmed using Trimmomatic (Bolger et al., 2014) and mapped to the NZ15MR0322 chromosome using Snippy (Version 3.1; https://github.com/tseemann/snippy). This generated a consensus MRSA0322

chromosome containing variant sites and indels and the modified chromosomes (each with 2,839,203 bases) were concatenated into a single MultiFASTA file. Gubbins (Croucher et al., 2015) was used to identify recombination. Three major regions were identified: (1) a ~123kb region previously identified by Price et al., of about 120kb in length (Price et al., 2012); and (2) two additional regions encompassing insertion sites of the two phages (Figure 1). These regions were removed from the alignment, resulting in an alignment of 2,138,149 coding bases, with 6,654 variable sites. A ML tree was produced using IQTREE (www.iqtree.org) with the following command:

```
iqtree -m TEST -mset JC,HKY,GTR -pre model_test -s cc398_parts123_recomb_filtered_core.fasta -nt 16
```

This phylogeny was used to assess the correlation between branch length and year of isolation with TempEst (version 1.5.4) (Rambaut, 2016) for signs of a temporal signal. Our sample spanned 22 years, with the oldest isolates sampled in 1993 and the youngest sampled in 2015. After removal of the regions highlighted by Gubbins, an examination of the output in TempEst suggested a strong temporal signal, with a MRCA in 1957. Using LSD (To et al., 2016) on the same tree (data not shown), we obtained the date to the MRCA of 1951 (95% CI obtained by bootstrapping: 1949 -1952), using the two pass mode. This indicated to us that there was sufficient heterochronicity in the data to undertake subsequent analysis using BEAST2.

The above alignment was then used as input to BEASTIFY, a Python script that produces NEXUS files suitable for input in BEAUTI 2.4.3 to produce XML files for BEAST 2.4.3. BEASTIFY takes as input the MultiFASTA alignment, and a Genbank file containing the annotation for the chromosome. It then partitions each site in the alignment into first, second, and third codon positions, inter-genetic regions, and sites with overlapping CDS annotations (i.e., sites belonging to more than one codon position). BEASTIFY allows the user to control which partitions are outputted, and whether to output

all bases or a random sample of bases from each partition. In our analysis, we only outputted first, second, and third codon positions.

Four models were trialled in BEAST 2.4.3: (1) Strict molecular clock (SMC) with a constant population coalescent prior on the genealogy; (2) SMC with an exponential population growth coalescent prior on the genealogy; (3) Uncorrelated Log-Normal Clock (ULNC) with a constant coalescent prior on the genealogy; and (4) ULNC with an exponential population growth coalescent prior on the genealogy. We set a log-normal prior on the clock rate with mean of -10 and standard deviation of 2.0, which established that 95% of the prior density on the clock rate was between $9.01e^{-7}$ and $2.29e^{-3}$. We assumed a GTR+Γ for each of the partitions, with the Γ distribution approximated by four categories. The α parameter of the Γ distribution was estimated from the data, as well as the frequency of each of the six possible substitutions. All other priors were kept as default.

Each model was run for 50 million iterations, and the traces were examined for convergence. The first 25 million steps were discarded as burn-in for all analyses. RWTY (https://github.com/danlwarren/ RWTY) was used to examine the posterior distribution of trees, and posterior distribution of split frequency. AICm was used to evaluate the model choice, as described by Baele et al.(Baele et al., 2013). The AICm suggested that the ULNC with an exponential population growth coalescent prior on the genealogy fitted the data better than any other model. The final results from this model all had effective sample sizes (ESS) of over 200 and had convergence across all runs. . The tree files for each run were combined and sub-sampled using logCombiner in order to produce 25,000 posterior genealogies, and to produce the final most recent common ancestor (MRCA) estimates. The maximum clade credibility tree (with nodes at median heights) was produced using TreeAnnotator.

REFERENCES

AGNOLETTI, F., MAZZOLINI, E., BACCHIN, C., BANO, L., BERTO, G., RIGOLI, R., MUFFATO, G., COATO, P., TONON, E. & DRIGO, I. 2014. First reporting of methicillin-resistant Staphylococcus aureus (MRSA) ST398 in an industrial rabbit holding and in farm-related people. *Vet Microbiol,* 170**,** 172-7.

ALIKHAN, N. F., PETTY, N. K., BEN ZAKOUR, N. L. & BEATSON, S. A. 2011. BLAST Ring Image Generator (BRIG): simple prokaryote genome comparisons. *BMC Genomics,* 12**,** 402.

BAELE, G., LI, W. L., DRUMMOND, A. J., SUCHARD, M. A. & LEMEY, P. 2013. Accurate model selection of relaxed molecular clocks in bayesian phylogenetics. *Mol Biol Evol,* 30**,** 239-43.

BANKEVICH, A., NURK, S., ANTIPOV, D., GUREVICH, A. A., DVORKIN, M., KULIKOV, A. S., LESIN, V. M., NIKOLENKO, S. I., PHAM, S., PRJIBELSKI, A. D., PYSHKIN, A. V., SIROTKIN, A. V., VYAHHI, N., TESLER, G., ALEKSEYEV, M. A. & PEVZNER, P. A. 2012. SPAdes: a new genome assembly algorithm and its applications to single-cell sequencing. *J Comput Biol,* 19**,** 455-77.

BOLGER, A. M., LOHSE, M. & USADEL, B. 2014. Trimmomatic: a flexible trimmer for Illumina sequence data. *Bioinformatics,* 30**,** 2114-20.

CROUCHER, N. J., PAGE, A. J., CONNOR, T. R., DELANEY, A. J., KEANE, J. A., BENTLEY, S. D., PARKHILL, J. & HARRIS, S. R. 2015. Rapid phylogenetic analysis of large samples of recombinant bacterial whole genome sequences using Gubbins. *Nucleic Acids Res,* 43**,** e15.

PRICE, L. B., STEGGER, M., HASMAN, H., AZIZ, M., LARSEN, J., ANDERSEN, P. S., PEARSON, T., WATERS, A. E., FOSTER, J. T., SCHUPP, J., GILLECE, J., DRIEBE, E., LIU, C. M., SPRINGER, B., ZDOVC, I., BATTISTI, A., FRANCO, A., ZMUDZKI, J., SCHWARZ, S., BUTAYE, P., JOUY, E., POMBA, C., PORRERO, M. C., RUIMY, R., SMITH, T. C., ROBINSON, D. A., WEESE, J. S., ARRIOLA, C. S., YU, F., LAURENT, F., KEIM, P., SKOV, R. & AARESTRUP, F. M. 2012. *Staphylococcus aureus* CC398: host adaptation and emergence of methicillin resistance in livestock. *MBio,* 3.

RAMBAUT, L., DE CARVALHO & PYBUS 2016. Exploring the temporal structure of heterochronous sequences using TempEst. . *Virus Evolution*.

SEEMANN, T. 2014. Prokka: rapid prokaryotic genome annotation. *Bioinformatics,* 30**,** 2068-9.

TO, T. H., JUNG, M., LYCETT, S. & GASCUEL, O. 2016. Fast Dating Using Least-Squares Criteria and Algorithms. *Syst Biol,* 65**,** 82-97.
